# The interplay between transcription and mRNA degradation in *Saccharomyces cerevisiae*

**DOI:** 10.15698/mic2017.07.580

**Published:** 2017-07-03

**Authors:** Subhadeep Das, Debasish Sarkar, Biswadip Das

**Affiliations:** 1 Department of Life Science and Biotechnology, Jadavpur University, Kolkata, India.; 2 Present Address: Laboratory of Molecular Genetics, Wadsworth Center, New York State Department of Health, Albany, NY 12201-2002, USA.

**Keywords:** promoter, mRNA degradation, transcription, functional coupling, Rpb4/7, SWI5, CLB2, RPL30, mRNA mark, coordinator

## Abstract

The cellular transcriptome is shaped by both the rates of mRNA synthesis in the nucleus and mRNA degradation in the cytoplasm under a specified condition. The last decade witnessed an exciting development in the field of post-transcriptional regulation of gene expression which underscored a strong functional coupling between the transcription and mRNA degradation. The functional integration is principally mediated by a group of specialized promoters and transcription factors that govern the stability of their cognate transcripts by “marking” them with a specific factor termed “coordinator.” The “mark” carried by the message is later decoded in the cytoplasm which involves the stimulation of one or more mRNA-decay factors, either directly by the “coordinator” itself or in an indirect manner. Activation of the decay factor(s), in turn, leads to the alteration of the stability of the marked message in a selective fashion. Thus, the integration between mRNA synthesis and decay plays a potentially significant role to shape appropriate gene expression profiles during cell cycle progression, cell division, cellular differentiation and proliferation, stress, immune and inflammatory responses, and may enhance the rate of biological evolution.

## INTRODUCTION

Gene expression in the eukaryotes is a highly complex process, involving a multitude of activities at different cellular compartments. Each of these processes was discovered independently and was subsequently investigated in an isolated fashion. Several current studies, however, revealed complex networks among them with additional layers of control as exemplified by the fascinating connections among various nuclear events, such as transcription elongation, capping, splicing, 3’-end processing, and nuclear export 1,2,3,4,5,6,7. Similarly, other independent studies uncovered many functional linkages among diverse cytoplasmic processes, such as translation, transport, and targeting of specific mRNAs to the sub-cytoplasmic location(s), their degradation, and the tight regulation exercised at each step. Although the nuclear mRNA biogenesis events were known to have far reaching effects on the cytoplasmic fate of the messages, the direct functional integration and cross-talk between the nuclear and cytoplasmic phases of gene expression and mRNA life were neither addressed nor actively investigated until lately. Some recent studies carried out in the baker’s yeast *Saccharomyces cerevisiae* revealed that transcription of a specific subset of genes and the stability of their corresponding mRNAs in the cytoplasm is intimately connected through various mechanisms 8,9,10,11,12,13,14. These studies demonstrated that the promoter, its associated *cis*-regulatory elements and special transcription factors, which bind to these elements, collectively affect the stability and decay rates of their corresponding messages and, thereby, functionally integrate the nuclear phase of mRNA life with their cytoplasmic fate 8,9,10,11,12,13,14. In this review, we focus on these studies carried out primarily in baker’s yeast and discuss the diverse nature of connections between the mRNA stability in the cytoplasm and the promoter/transcriptional activities of the corresponding genes in the nucleus. Also, we extend the issue of possible interplay between mRNA synthesis and degradation in the mammalian system by presenting several preliminary and speculative findings. Finally, the contribution of the functional coupling between the mRNA synthesis and decay process is discussed which plays vital roles during cell division/cycle, stress response, cellular differentiation, cell-cycle progression, cell proliferation, immune and inflammatory response, viral infection as well as in facilitating evolution.

## THE BIRTH AND EARLY LIFE OF THE mRNAs: BIOGENESIS IN THE CELL NUCLEUS

**Figure 1:**
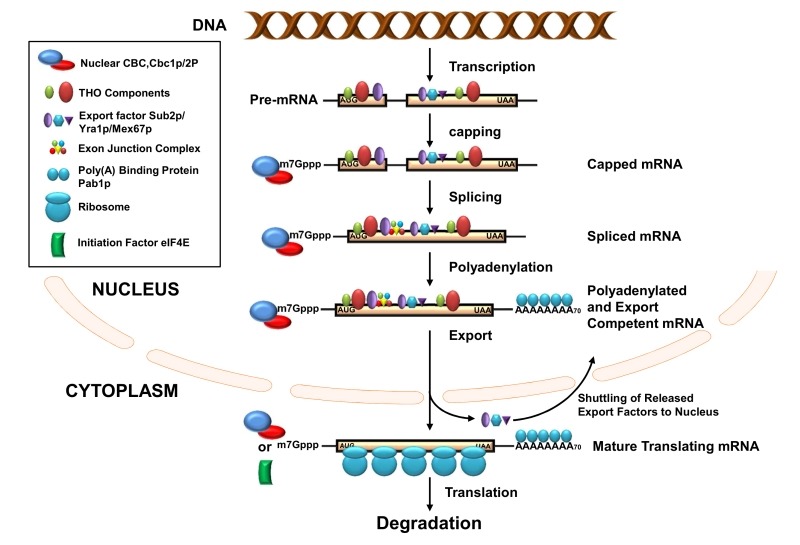
FIGURE 1: mRNA life-cycle in eukaryotic cells. Schematic view of the nuclear and cytoplasmic phases of the mRNA life cycle. Various mRNPs which are recruited onto/associated with the maturing transcripts during different stages are schematically indicated by solid colored symbols. Symbols are either annotated directly or denoted in the associated legend box. THO components/maturing factors/mRNA-binding proteins are released from mRNA once the mRNA matures and becomes export-competent. Similarly, export factors are also released from the transcript body once the mRNA arrives at the cytoplasm and shuttle back into the nucleus. In the cytoplasm, mRNAs may remain associated either with nuclear CBC (while undergoing a pioneer round of translation) or with eIF4E (while undergoing subsequent steady state translation) which is indicated in the diagram. For simplicity, other mRNA binding proteins remaining associated with translating mRNAs are not shown except for CBC and eIF4E. AUG and UAA are indicating the beginning and end of the open reading frame (ORF) carried by the message.

In eukaryotes, productive expression of a gene involves transcription, maturation of the transcripts in the nucleus, their export into the cytoplasm, subsequent translation of the translation competent messages, and their destruction in specific cytoplasmic location. During this long journey, every transcript undergoes extensive covalent modification and structural remodeling events. Nuclear events of mRNA biogenesis involve the transcription by RNA polymerase II (RNAPII), capping of the primary transcript at the 5’-end, splicing, and maturation at the 3’-end of the message, involving a site-specific cleavage and polyadenylation 1,15,16,17,18,19,20,21,22,23,24 (Figure 1). During these modification events, every transcript is associated with a wide repertoire of mRNA-maturing factors and heterogeneous nuclear ribonucleo proteins (hnRNPs), which, in turn, determines the fate of the transcribing mRNAs. This dynamic RNA-protein interaction begins with the association of the heterodimeric nuclear cap binding complex (CBC) to the m7G cap located at the 5’-end of the nascent mRNA 25,26. Binding of the cap structure by CBC is followed by the recruitment of transcription/export (TREX) complex consisting of THO proteins (Hpr1p, Mft1p, Tho2p, Thp2p), mRNA export factors, RNA helicase Sub2p (UAP56 in human), and RNA binding protein Yra1p (REF/ALY in human). Deposition of the TREX complex onto the transcribing message facilitates both the splicing (if intron is present) and subsequent association of the transcript with the export receptor Mex67p, Mtr2p (NXF1:p15 in human), various hnRNPs (such as Gbp1p, Hrb1p, and Tex1p), and poly(A) tail binding protein Pab1p 27. Efficient and accurate splicing and 3’-end maturation leads to the recruitment of the exon junction complex (EJC) onto the newly formed exon-exon junction and poly(A) tail binding proteins Pab1p (PABP in human) onto the polyadenylated tail, respectively. The collective and concerted action of the whole spectrum of RNA-binding proteins (RBPs) ultimately leads to the formation of mature export-competent mRNPs 1,15,16,17,18,19,20,21,22 (Figure 1). These export-competent mRNPs are then released from the transcription site at the chromatin and gradually move through the inter-chromatin space to the nuclear periphery where they dock at the nuclear pore complexes (NPC) 1. Once the mature mRNPs successfully dock at the NPC, they passage through the NPC structures and are finally released into the cytoplasm. Nuclear export thus represents the culmination of the nuclear phase of gene expression, which is regarded as the early life of mRNPs 28,29,30,31.

In *S. cerevisiae*, transcription and nuclear pre-mRNA processing events are physically and functionally coupled via the C-terminal domain (CTD) of the Rpb1p (largest subunit of RNAPII) 24,32,33,34,35. The CTD acts as a loading platform for transcription and other mRNA processing factors 27,29,34,36,37,38,39. Each event of the mRNP biogenesis is thus believed to impact its following step, depending on the status of the preceding event(s). Functional interplay was demonstrated (i) to enhance the probability of the formation of export-competent and productive mRNPs and (ii) to reduce the possibility of generating functional defective transcripts. The functional coupling thus lowers the risk of forming unproductive polypeptides and minimizes the requirement of quality control steps 27,29,34,36,37,38,39. Defective/faulty messages still arise, despite having a tight functional coupling between various nuclear mRNA biogenesis events.However, they are rapidly eliminated by a variety of mRNA surveillance and quality control mechanisms 2,40,41,42,43,44,45,46,47,48,49,50,51.

## THE LATE LIFE AND DEATH OF mRNAs: mRNA TRANSLATION AND DEGRADATION IN CYTOPLASM

In the cytoplasm, the mRNPs undergo another remodeling event to shed off some of the mRNA-binding proteins carried from the nucleus. A unique pioneer round of translation immediately follows this remodeling event while they still carry the nuclear CBC 52,53. This distinct round is used to detect any potential in frame premature termination codon (PTC) in the translating message. If such a PTC is detected, it is promptly targeted for degradation by the non-sense mediated decay (NMD) pathway to avoid production of truncated proteins 54. Messages lacking a PTC survive the NMD action and subsequently undergo another mRNP remodeling, which involves the exchange of the nuclear CBC at the 5’-cap of mRNA with the translation initiation factor eIF4F (consisting of eIF4E and eIF4G) 54. The remodeled message is then engaged in the steady state of productive translation to produce the cellular pool of proteins 52,53. Notably, several exported mRNPs, however, do not enter the regular translation cycle and, instead, are transported to several special cytoplasmic locations (e.g. stress granules) for future use 55,56.

After predestined rounds of translation a translating mRNA is transformed into a degradation-committed message and eventually disengages from translating polysomes to associate with the cytoplasmic P-bodies or stress granules, which are considered the cellular sites of mRNA decay in *S. cerevisiae*. This degradation-committed message is subsequently destroyed by the default degradation pathway within the P-bodies 40,57,58. Default degradation of mRNAs is a highly regulated process that is governed by a distinct set of genes and plays a vital role to dictate the basal steady state level of all mRNAs, which, in turn, determines the total cellular pool of proteins 40,59,60,61. Defective and aberrant mRNAs, intragenic, intergenic, promoter-associated RNAs (transcriptional noise), anti-sense RNAs (generated for example as regulatory RNAs) and other byproducts of gene expression (excised introns, external or internal spacers, etc.) are eliminated in a regulated manner by another set of selective/specialized mRNA degradation processes termed mRNA surveillance mechanisms 40,62,63,64,65,66,67,68. In this article, we will principally focus on the default mRNA degradation mechanism due to its relevance to the current topic. Readers are suggested to consult following review articles for in-depth discussion of the diverse cellular roles of mRNA degradation and mRNA surveillance 2,40,41,42,43,44,45,46,47,48,49,50,51.

**Figure 2:**
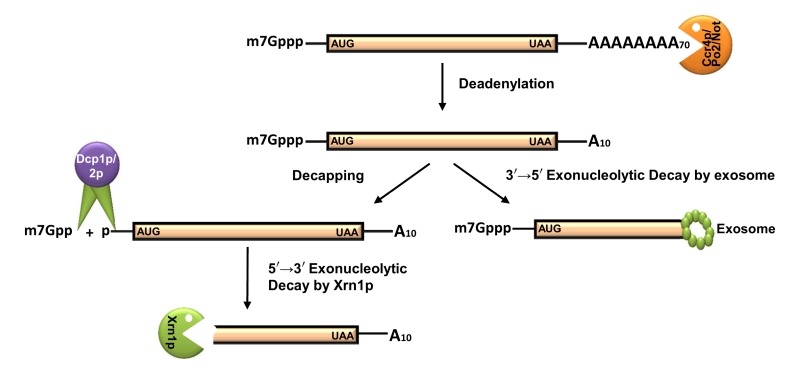
FIGURE 2: Default pathway of mRNA degradation in *S. cerevisiae*. Almost all mRNAs undergo decay by the deadenylation-dependent pathway. Thereby, the poly(A) tail is gradually and progressively shortened by the deadenylase activity of the Ccr4/Pop2/Not complex. Following deadenylation, the mRNA can be degraded by one of two mechanisms. The major mechanism involves decapping by Dcp1p/2p, following a 5’→3’ decay by Xrn1p. The minor mechanism includes a 3’→5’ decay by the cytoplasmic exosome and Ski7p. AUG and UAA are indicating the beginning and end of the ORF carried by the message. Only relevant decay components are shown by annotated symbols. Proteins which remain associated to translating/degrading mRNAs during different stages of decay are not shown.

The default decay process is initiated with the shortening of the poly(A) tail from 60-90 residues long adenylate tail (300-400 residues in mammalian mRNAs) to a 10-15 residue oligo-A state 40,59,60,61. This step, called deadenylation, is catalyzed either by the Ccr4p/Pop2p/Not complex (major deadenylation machinery) or by the Pan2p/Pan3p complex (a subsidiary deadenylation machinery) 6,69 (Figure 2). Deadenylation is followed by the removal of the 5’-cap structure by the concerted action of the decapping complex consisting of Dcp1p/Dcp2p, which is catalytically stimulated *in vivo* by Pat1p, Edc1-3p, Scd6p, the Lsm1-7p complex, and the DEAD box helicase Dhh1p 40,70,71,72,73,74. The decapping reaction exposes the 5’-monophosphate of the terminal residue and subsequently promotes the degradation of the transcript body in a 5’→3’ direction by the major cytoplasmic exoribonuclease Xrn1p 75. Alternatively, the degradation may also proceed in a 3’→5’ direction right after the deadenylation step by the cytoplasmic exosome and the Ski complex 76,77,78 (Figure 2). Processive degradation of the mRNA transcript body from the 3’→5’ direction results in the formation of a left-over residual oligonucleotide structure with the 5’-cap, which is eventually removed by DcpS 79.

## CONNECTING BIRTH TO DEATH: COUPLING TRANSCRIPTION AND mRNA DECAY IN YEAST

Emerging studies carried out in *S. cerevisiae* implicated various mechanisms that functionally couple nuclear transcription and cytoplasmic mRNA stability/decay. The majority of these mechanisms are mediated by a variety of *trans*-acting protein factors (collectively termed "coordinators") that are selectively recruited onto specific subsets of transcripts by distinct *cis*-acting sequences (such as gene promoters). Recruitment of the coordinator "marks" the specific subset of transcripts and thereby alters their stability in response to a physiologic cue. These "marks" on the transcript are later decoded in the cytoplasm by a variety of mechanisms, which ultimately stimulates specific mRNA decay factors and thereby triggers the kinetics of decay of a specific or a cluster of transcripts in response to various cues. Functional coupling modulated by different classes of coordinators are discussed below.

### Functional Coupling mediated by RNAPII core proteins

Two subunits of the core RNAPII, Rpb4p and Rpb7p, were implicated in the functional coupling between transcription and mRNA degradation 12,13,14,80. The first clue about their functional role in linking transcription to mRNA decay came from the observations that (i) these two subunits independently form a Rpb4/7p heterodimer and (ii) the stoichiometry of the heterodimer in the cell to the other subunits of the RNAPII holoenzyme substantially deviates from the unity 81. Moreover, Rpb4p/7p subunits were frequently found to dissociate from RNAPII 82,83. Consistent with these findings, these two subunits were found to participate in the co-transcriptional recruitment of the 3’-end processing factors and appropriate usage of polyadenylation sites 84, mRNA export 85, and translation/mRNA decay 12,13,14,80. A potential cytoplasmic function of Rpb4/7p proteins was further suggested from the observation that they are able to shuttle between nucleus and cytoplasm in a transcription-dependent manner 86.

**Figure 3:**
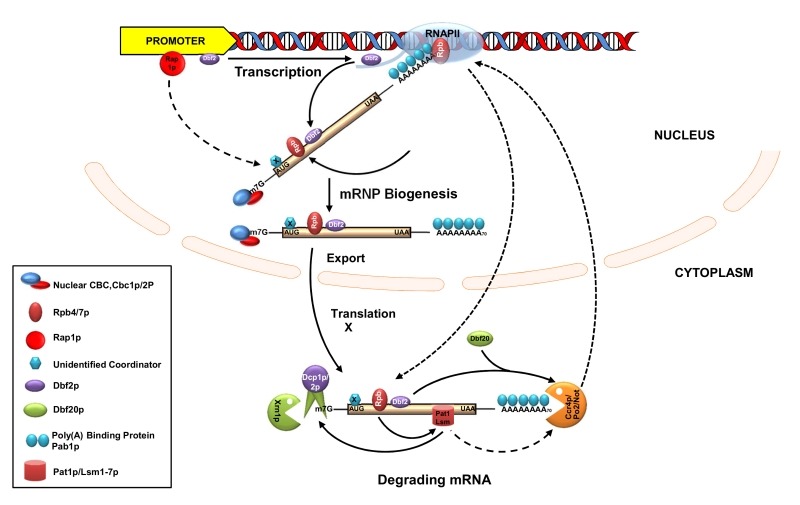
FIGURE 3: The interplay between the transcription and mRNA degradation in *S. cerevisiae*. Schematic diagram showing the functional coupling between the mRNA synthesis and degradation. Functional coupling is achieved by marking of the transcribing and maturing messages by various coordinators either through the Rpb1-CTD of RNAPII in a transcription-dependent manner (such as those of Rpb4/7p and Dbf2p) or in a transcription factor (Rap1p)/promoter-dependent manner (such as that of a hitherto unidentified coordinator(s), coded X). The effect of various elements on the recruitment of diverse coordinators is indicated either by the solid (demonstrated) of dashed (postulated) arrow. Export-competent mRNPs undergo translation after arriving in the cytoplasm and are subsequently degraded via the general default decay pathway. During this stage, the Rpb4/7p dependent recruitment of Pat1/Lsm1-7p and influence of Dbf2p together with Dbf20 on the CCR4/NOT complex to further stimulate decay via the activation of other decay components are indicated by the solid arrow. Note that the demonstrated influence of Pat1/Lsm1-7p on the decapping complex is shown by the solid arrow, whereas its potential but questionable impact on CCR4/NOT is indicated by a dashed arrow. Mutual influence of each process on the other is indicated by dashed arrows. Only relevant components are shown by annotated symbols. Other proteins which remain associated to translating/degrading mRNAs (such as eIF4E) during translation and decay are not shown.

Several pieces of information provided evidence that the Rpb4/7 heterodimer promotes the decay of a specific class of cellular mRNAs encoding ribosomal proteins and translation factors (collectively designated PBF, protein biosynthetic factors) 12,13. First, both Rpb4p and Rpb7p, affect the deadenylation step of the decay process of these mRNAs. Second, both of them were found to interact with the mRNA decapping components of the Pat1p-Lsm1-7p complex 12,13. Third, together with Lsm1-7p and Dcp1p, Rpb4/7p was found to localize to cytoplasmic P-bodies, the site of mRNA decay in the cell (see above) 12,13. Fourth, a mutation in either of these proteins led to an alteration of the number of P-bodies in the cell. Collectively, these findings are consistent with a model in which Rpb4/7p may recruit Pat1p to the substrate mRNA and, thereby, may affect mRNA degradation via its interaction with Pat1p 3 (Figure 3). Notably, Pat1p was demonstrated to be a hub of other mRNA decay factors and is also required for recruiting Lsm1-7p, Dcp1/2p, and Xrn1p to the mRNA 87,88. Interestingly, the functions of Rpb4p/7p heterodimer in the process of coupling transcription and RNA decay largely depends on the previous association of the Rpb4/7p heterodimer with the RNAPII holoenzyme 14,80. Although this model is very attractive and provocative, the mechanistic insight of how they impact the mRNA deadenylation is not very clear because neither a direct interaction between Rpb4/7p and the deadenylase complexes Ccr4p/Pop2/Not/Pan2-3p was detected 12,13 nor could Pat1 be linked to deadenylation 89. Recently, an interaction between Rpb4/7 with the translation initiation factor eIF3 was suggested to be responsible for the observed deadenylation 14, although having no mechanistic insight regarding this connection.

### Functional coupling mediated through the specific gene promoters

An early instance of the influence of the specific promoter on the decay of its cognate message was exemplified by a study in mammalian cells in which the abundance of the β-globin mRNA was demonstrated to be affected by its own promoter 11. In this investigation, the abundance of a mutated β-globin mRNA, harboring an in frame PTC expressed from a β-globin promoter in Hela cells, was found to be markedly low compared to that of a WT β-globin message. This observation suggested that the PTC-containing β-globin mRNA was undergoing a rapid decay, owing to the presence of the PTC when expressed from its native promoter 11. Remarkably, when the native β-globin promoter was replaced with a viral HSV-Tk promoter, the decreased abundance of mutated PTC globin mRNA was rescued, thus indicating a diminished degradation of the mutated β-globin message under the control of the viral promoter 11. Using the primer extension analysis, they also demonstrated that the utilization of the transcription site of the mutant message remained identical in both cases. This finding ruled out the possibility of the existence of any altered 5‘-untranslated region (UTR) of the β-globin transcript expressed from the viral promoter, which could have otherwise altered its stability. This finding, therefore, marked the first demonstration of the influence of a native promoter on the abundance and stability of its cognate message. However, the investigators were unable to gain any insight into the mechanism of the altered stability of the β-globin transcript expressed under different promoters 11.

More recent studies in the baker’s yeast *S. cerevisiae* demonstrated that promoters and associated *cis*-regulatory elements of certain genes and their cognate transcription factors play crucial roles in coupling their transcription and the decay rates of their corresponding transcripts 8,9,10,11,90. One instance of such functional influence of the promoter and the associated upstream activating sequence (UAS) affecting the mRNA decay is provided by the *RPL30* mRNA, encoding the large ribosomal protein 30 in *S. cerevisiae*. Promoter swapping experiments showed that exchanging the native UAS of the *RPL30* gene with that of the *ACT1* gene, without altering its coding sequence, has a remarkable influence on the stability of the *RPL30* mRNA 9. Stability of the *RPL30* transcript expressed from its native promoter (harboring *RPL30* UAS) vs. the *ACT1* promoter (harboring *ACT1* UAS) displayed a dramatic difference 9. Further analyses uncovered that the *RPL30* promoter harbors two binding sites for the specific transcription activator Rap1, and eliminating them led to the dramatic stabilization of the *RPL30* mRNA. Thus, recruitment of Rap1p to the *RPL30* UAS appears critical for the stimulation of the decay of the corresponding message (Figure 3). Therefore, this observation directly connected the transcription factor Rap1p to the promoter-influenced decay kinetics of the *RPL30* mRNA. Consequently, Rap1p has been termed a "synthegradase" to underscore its effect in coupling transcription with mRNA decay, presumably by marking the *RPL30* message 3,9 (Figure 3). However, it is still unknown which factor is marked on the *RPL30* message to affect its decay. An extension of this work by Dori-Bachash *et al*. 8 provided further evidence that swapping the upstream *cis*-regulatory sequences of orthologous genes from two related yeast species affects both, the mRNA transcription and the decay for some genes 8. Notably, adjacent yeast genes sharing a common promoter displayed similar mRNA decay profiles, thus indicating the existence of a pervasive promoter which mediates the coordination between transcription and mRNA decay in yeast 8. Remarkably, transcription also appears to be coupled to the process of decay for same sets of mRNAs in mouse and humans 8.

By using a powerful and sensitive single-cell single-molecule FISH technique, another independent study demonstrated that the promoters of the *SWI5* and *CLB2* genes in *S. cerevisiae* are playing a vital role in modulating the stability of their corresponding messages in a cell cycle-dependent manner 10. *SWI5* is a transcription regulator associated with late mitosis genes, and *CLB2* is a G2 phase cyclin that promotes the entry of yeast cells into mitosis. Replacing the *SWI5* and *CLB2* promoters with the *ACT1* promoter altered the native decay rates of these two mRNAs. This regulation involves the mitotic exit network (MEN) kinase Dbf2p and its interacting partner polo kinase Cdc5p 91, as well as the major cytoplasmic deadenylase the Ccr4p/Pop2p/Not complex. Their finding is consistent with a model where Dbf2p is first recruited to the *SWI5* and *CLB2* promoter, subsequently loaded onto these messages in a transcription-dependent manner and eventually carried to the cytoplasm (Figure 3). Once in the cytoplasm, Dbf2p is associated with the ancillary factor Dbf20p (assists Dbf2p function and displays a synthetic-lethality with it, 92) at the onset of pro-metaphase to metaphase transition, thereby coordinating the timing of their decay 10 (Figure 3). Remarkably, Dbf2p interacts with the Ccr4p/Pop2/Not complex 93 and thereby promotes their degradation 10. However, it was not clear how Dbf2 is recruited to the *SWI5* and *CLB2* promoters and, subsequently, onto these messages to influence their cytoplasmic fate. Nevertheless, the study revealed that Dbf2, the mitotic kinase, acts as a "coordinator" and thereby connects the transcription with the mRNA decay 10.

Expression of the *GAL* genes in *S. cerevisiae* provides another example for the promoter-assisted decay of mRNAs. The addition of galactose to yeast cells growing in a medium containing raffinose or another non-fermentable carbon source leads to a rapid and huge transcriptional activation of the *GAL* genes. This transcriptional burst is rapidly attenuated by the addition of glucose and accompanied by the selective decay of the *GAL* and other associated messages 94,95,96. Notably, the glucose-induced decay of the *GAL* transcripts requires the native promoters of the *GAL* genes since replacement of the native *GAL7* promoter by a constitutive *ADH1* promoter led to a diminished decay of the *GAL7* transcripts under the same condition 97. Consequently, the enhanced stability (diminished decay) of the *GAL7* transcripts expressed from the *ADH1* promoter could be attributed to the promoter itself that harbors the binding sites for the transcription factor Rap1p, which was found to be linked to the stability of other transcripts (see above) 9. However, the exact mechanism how Rap1p brings about the alteration of the stability of the *GAL7* transcripts is unclear.

In a recent study, Snf1p, the yeast ortholog of mammalian/human AMP-activated protein kinase involved in multiple and diverse stress conditions 98,99,100,101,102, was also found to govern the glucose-induced decay of mRNAs 103,104. Snf1p has both, a direct and an indirect role in stress response (i) by substrate-level phosphorylation 105,106 and (ii) via its involvement in the transcriptional control of gene expression 107, respectively. Remarkably, compromising the activity of Snf1p led to the rapid destabilization of Snf1p-dependent transcripts 105. Conversely, if Snf1 is constitutively active (such as in a *reg1* yeast mutant), the same sets of transcripts were found to undergo diminished mRNA decay 96,105. In low glucose concentration, Snf1 activates the transcription of glucose-induced genes required for energy metabolism in the absence of glucose. Conversely, when glucose concentration is high, Snf1 activity is inhibited and the level of glucose-induced transcripts rapidly drops off. This sharp decline in mRNA levels is due to a termination of transcription and a stimulation of their decay rate, thus appearing to couple transcription to mRNA degradation 104,105. In their studies, Braun *et al*. demonstrated that fusing nonglucose-responsive genes *MAP2* and *IDP2* to the *ADH1* promoter caused a dramatic destabilization of these corresponding non-cognate transcripts, thereby showing that the *ADH1* promoter alone could modulate glucose-induced mRNA decay 103,104. Binding sites of a specific transcription factor, Adr1p, in the *ADH1* promoter appeared to be a crucial factor for the glucose-induced mRNA decay. Although the exact mechanism how Snf1p brings about the decay of the glucose-induced messages remained unclear, an involvement of additional RNA binding proteins, such as Vts1p, was postulated. In this respect, it is very interesting to note that four mRNA decay factors, Eap1p and Ccr4p (both interacting with Vts1p), Dhh1p and Xrn1p were found to be targets for Snf1p-dependent phosphorylation (note that Snf1p is AMP-activated protein kinase) as revealed by a previous phosphoproteomic study 105. Consequently, the deletion of *XRN1*, *DHH1* or *CCR4* led to the enhanced stability of Snf1-dependent transcripts. Moreover, Puf5p, another target of Snf1p, was found to promote mRNA degradation by recruiting the Ccr4p/Pop2p/Not complex along with the helicase Dhh1p and the decapping enzyme Dcp1p to promote deadenylation, decapping, and decay 108,109,110. Therefore, Snf1p-dependent transcription and decay of glucose-specific subsets of mRNAs were proposed to be stimulated by triggering the cytoplasmic decay factors, possibly via the modulation of marked Vts1p 104. However, further investigation is required to dissect the exact mechanism of Snf1-dependent, glucose-induced, transcription-coupled mRNA decay.

### Functional coupling mediated by mRNA decay factors

Apart from promoters and transcription factors, three mRNA decay components, the major deadenylase, Ccr4p/Pop2p/Not complex, the principal decapping factor, Dcp2p, and the major cellular exoribonuclease Xrn1p, are also implicated in the process of transcription. Ccr4p/ Pop2/Not, a nine subunit protein complex in *S. cerevisiae* constitutes the predominant deadenylase which catalyzes the initial deadenylation step of polyadenylated mRNAs prior to their decapping by the Dcp1p/Pat1p/Lsm complex (see the previous section) 111,112,113,114. This complex consists of two functional 3’→5‘ exonuclease subunits, Ccr4p (the major catalytic subunit), and Pop2p/Caf1p (an ancillary catalytic subunit). In addition, the complex contains Not1p-5p (Not1p is a large scaffolding protein), Caf40p and Caf130p (two accessory proteins) 112,114. Ccr4p constitutes the major exonuclease (a member of the ExoIII exonuclease family) 111,113, which functionally interacts with the Pop2p exonuclease (a member of RNase D family) 115. Despite the presence of Pop2p in the complex, Ccr4p plays the central role in the deadenylation event. Not1p, Caf40p, and Caf130p were suggested to participate in the adaptation of the deadenylase complex to diverse cellular mRNA pools via the interaction with various regulatory proteins 113.

Remarkably, for a long time the involvement of Ccr4p in the deadenylation process remained unnoticed, and the protein was initially discovered as a transcription activator of alcohol dehydrogenase II and some glucose-regulated genes 116,117, much before its functional role as deadenylase was revealed 111,112,113,114. Other independent studies uncovered the functional implication of Not proteins in the repression of transcription in TATA-less promoters 118,119,120. Additional genetic and physical interactions of Not proteins with a broad spectrum of various promoters and transcription-associated factors were subsequently uncovered 121,122,123. Furthermore, the Ccr4p/Pop2/Not complex was also found to be involved in transcription elongation 124,125,126. Thus, the functional involvements of the Ccr4p/Pop2/Not complex in both, the transcription and mRNA degradation, are suggestive of a strong functional interplay between them. However, the underlying mechanistic aspects of these connections and their associated functional significance are not yet completely understood. Further investigations are necessary to unveil the cross-talks between mRNA synthesis and degradation dependent on the Ccr4p/Pop2/Not complex.

The second example of functional influence on the process of mRNA transcription by a decay component is provided by Dcp2p, which is the major catalytic subunit of yeast decapping complex and belongs to the nudix family of pyrophosphatase 127,128. Dcp2p cleaves the cap structure and thereby releases m7-GDP and 5‘-monophosphate mRNA 72. Strikingly, the Dcp2 protein has an extended CTD, which is not essential for general mRNA decapping. Consistently, it was shown to shuttle into the nucleus 129, and its extended CTD was reported to have functional sites that may trigger transcription 130. Although the exact mechanistic details of nuclear shuttling and functional involvement of Dcp2p in transcriptional regulation is not clear, it is believed that this extended region plays some functional role in controlling mRNA transcription 131.

Xrn1p, the major cytoplasmic 5’→3‘ exoribonuclease, presents another instance of the influence of an mRNA decay factor on the nuclear transcription 132,133. As mentioned above, Xrn1p is a nonessential and highly conserved cytoplasmic 5’→3‘ exoribonuclease which degrades global cytoplasmic transcripts with a free 5‘-monophosphate end resulted from a decapping reaction or from endoribonucleolytic cleavage 4. Notably, *xrn1* mutants display multiple phenotypic defects, such as sensitivity to Li^2+^ ions 98, and deficiency in genetic recombination, meiosis, and telomere maintenance 134. Two recent studies presented evidence of the functional role of Xrn1p in connecting the transcription with mRNA degradation in baker’s yeast 132,133. The first study by Haimovich *et al*. proposed a stimulatory role of Xrn1p in transcription by showing that deletion of *XRN1* led to a reduced transcription rate as measured by nuclear run-on assay, FISH (to show that a decreased number of mRNA in the transcription sites of the affected gene is a consequence of the loss of Xrn1p) and global gene expression analyses 133. The study by Sun *et al*., in contrast, inferred that depletion of Xrn1p caused a global enhancement of mRNA synthesis rates as measured by incorporation of 4-thiouracil 132. Remarkably, despite the perceptible disagreement, both studies concluded that Xrn1p plays a crucial role in "buffering" the global cellular mRNA levels in response to alterations in either synthesis or mRNA decay. Thus, while the actual role of Xrn1p in coupling transcription with mRNA degradation is controversial, the "buffering" effect of this protein to maintain cellular mRNA level appears consistent and acceptable. Further studies are essential to (i) resolve the apparent contradictions between the findings of these two studies and (ii) throw light on the mechanism involved in Xrn1p dependent "buffering" of mRNA level and coupling of mRNA synthesis and decay.

The functional influence of mRNA decay factors on transcription is further supported by the following additional observations. First, null strains of yeast in the *DCP1* gene (Δ*dcp1* strains) were observed to have decreased decay rates of specific reporter mRNAs without a similar enhancement in their cellular abundance. This finding thus indicated that the cells possess the capability of counteracting the decline in mRNA decay rates by appropriate alterations in their transcription rate 135. Also, strains depleted for the decapping activator Edc1 fail to trigger new transcription during a shift in carbon source 136. Thus, the collective evidences presented above strongly argue that the events of mRNA transcription and decay are coupled.

## COUPLING BETWEEN TRANSCRIPTION AND mRNA DECAY IN MAMMALS

Transcription and mRNA degradation also appears to be functionally coupled in mammalian cells. The very first instance of interconnection between mRNA synthesis and turnover was reported in the studies by Enssle *et al*. in mammalian cells, which demonstrated the dependence of the stability of a β-globin mRNA harboring a PTC on its native promoter (see above) 11. Subsequently, recent studies now present evidence of possible functional connections between transcription and mRNA degradation in diverse mammalian systems as well. The most direct support of interconnection between transcription and mRNA degradation rate in the mammalian system came from the studies by Dori-Bachash *et al*. 8. These authors noted a significant positive correlation between changes in the steady-state levels of mRNAs and degradation rates in induced pluripotent stem cells (iPS) while comparing the decay rates of global mRNAs and human foreskin fibroblasts (HEFs) (note that HEF is derived from iPS), which implied a functional connection between these opposing processes in these cells 8. Furthermore, they also compared the mRNA degradation rates of the human-mouse orthologous mRNAs using the mRNA decay datasets carried out in human B cells (BL41) and murine NIH-3T3 fibroblasts and found similar rates of decay of the orthologous messages. These findings collectively support the existence of a global coordination between transcription and mRNA degradation in the mammalian system.

Additional instances for the interplay between synthesis and decay of mRNA in mammalian cells are exemplified by several families of mammalian proteins, BTG/Tob, TIS11, and KSRP. Many members of these protein families play pivotal roles in both, nuclear transcription and cytoplasmic mRNA turnover 137,138,139. Mammalian BTG/Tob family of antiproliferative proteins consists of six members, BTG1, BTG2/PC3/Tis21, BTG3/ANA, BTG4/PC3B, Tob1/Tob and Tob2. They are involved in the control of cell cycle progression in a variety of cell types 139,140. Expression of BTG/TOB proteins leads to the inhibition of cell cycle progression 139,140,141,42, whereas loss of their expression is associated with lung, thyroid and breast tumors 143,144,145,146. Notably, all the members of this family are characterized by having a highly conserved N-terminal BTG domain (104 to 106 amino acid residues long) and a much less conserved C-terminal end 139,141,142,147,148,149,150. The BTG domain is implicated in the interaction with components of transcription as well as mRNA decay 139. Several members of the family promote binding of a diverse array of transcription factors to their respective target sites, thereby stimulating the transcription of corresponding genes. These include Hoxb9 (homeobox transcription factor) 151, TRα and Myogenic factor MyoD in myoblast cells, RARα, c-Jun, and Myogenin 152. One family member, BTG3, however, exerts its action reversely by inhibiting the binding of the transcription factor E2F1 to its target sequence and thereby affects the S-phase entry and cell cycle progression 153. Regardless of their mode of action these proteins are thought to regulate the DNA-binding activity of their target transcription factors by promoting chromatin remodeling of the appropriate genomic segments 151,153,154. Also, all BTG proteins (except for BTG4) promote mRNA deadenylation and turnover by interacting with CNOT7 and CNOT8 (a paralog of CNOT7) subunits of the Ccr4-Not complex in the cytoplasm 149,155,156,157,158,159,160. In an alternative mechanism, Tob1 and Tob2 stimulate mRNA turnover via their association with the poly(A)-binding protein PAPBC1 140,161,162. In agreement with their dual roles both in the transcription and mRNA turnover, BTG/Tob family members are present in the nucleus and in cytoplasmic P-bodies (enriched in decay-committed mRNA and mRNA decay factors) 161,163. Whether a given member would influence the transcriptional activation or promote mRNA turnover is believed to be dictated by their selective intracellular localization 139. However, no direct evidence for the involvement of these proteins in coupling transcription with decay is currently present.

The second protein family, TIS11, consisting of BRF-1, BRF-2, and TTP, are known for a long time as the effector of degradation of mRNAs harboring AU-rich elements (ARE) in their 3‘-UTR region, such as those encoding growth factors, inflammatory cytokines, and proto-oncogenes 164,165. Remarkably, two recent studies presented evidence for a decay-independent role of TTP in the NF-κB-dependent transcription by demonstrating that overexpression of TTP inhibits such transcription 166,167. Furthermore, the TTP-dependent inhibition of NF-κB-dependent transcription is independent of its RNA-binding ability 166,167, which is accomplished by two alternative mechanisms, (i) by interfering with the nuclear import of the p65 subunit of NF-κB, 167 and (ii) by recruiting histone deacetylases (HDACs) 168 to the genomic locus of the genes typically regulated by NF-κB 166. The KSRP family of RNA-binding proteins provides the third group of proteins which appear to bridge the transcription with mRNA decay 166. Members of this group play crucial roles in various aspects of mRNA metabolism, such as transcription, splicing, APOBEC mediated mRNA editing, mRNA localization, and mRNA decay in specific mammalian cell types 138. Moreover, all members of this family contain a proline-glycine (PG) rich region and a putative α-helical region in the extreme N-terminal end, followed by four KH domains in the central region, and four Y-rich (tyrosine) repeats in the C-terminal segment 138. Notably, KSRP proteins were recently found to exert pivotal influence in the transcriptional regulation of the c-myc gene by binding to an AT-rich element located 1.7 kb upstream of the c-myc promoter 169 which is facilitated by the N-terminal PG-rich α-helical and C-terminal Y-rich repeats to establish a specific association with TFIIH 138. Remarkably, current data also suggest that KSRP bind to the several mRNAs harboring ARE in their 3‘-UTR and thereby promote their regulated degradation 170,171,172. Consequently, further analyses had shown that these proteins recruit the cytoplasmic exosome and other decay factors onto their target messages 170,171,172. Remarkably, the decay-promoting activity of KSRP are regulated at the post-translational level by modulating the phosphorylation of a threonine (Thr692) or serine (Ser193) residue 173,174,175 which presumably impairs the decay-promoting activity of KSRP. It is believed that phosphorylation inhibits the binding of KSRP proteins either to their target RNAs or the exosome and other decay factors 173,174. Additionally, these proteins facilitate the nuclear maturation event of a cohort of microRNAs (miRNAs) by interacting with their precursors as well as with both Drosha and Dicer 176,177. The collective evidence thus indicates that members of TIS11 and KSRP families play a crucial role in both, transcription and degradation of a variety of transcripts.

Remarkably, a recent study strongly suggests the existence of a functional coupling between the transcription and mRNA degradation in the mammalian cell, which is harnessed by the gamma-herpes virus during its infection cycle 178. The gamma-herpes virus destabilizes at least a portion of the host transcriptome by encoding the viral endonuclease SOX and thereby facilitates endonucleolytic cleavage of host transcripts 179, which are subsequently cleaved by cytoplasmic exoribonuclease Xrn1 180. This accelerated decay of the specific cellular transcripts, in turn, strongly represses transcription rate of the corresponding genes, which is achieved by decreased RNAPII recruitment to the transcriptionally impacted cellular promoters 178. Furthermore, the repression requires the activity of cellular Xrn1, which suggests that the level of Xrn1 activity in the cytoplasm is critical to bring about the transcriptional alterations. Also, it was further demonstrated that the viral mRNAs somehow escape the degradation induced transcriptional repression, possibly due to the more efficient recruitment of unused cellular RNAPII to the viral promoters. Thus, gamma herpes viruses indeed seem to exploit the putative functional feedback loop between mRNA transcription and decay in mammalian cells 178.

The collective evidence, therefore, indicates the functional involvement of several mammalian protein families in the synthesis and degradation of a diverse set of specific mRNAs controlling cell differentiation, cell-cycle progression, cell proliferation, immune and inflammatory response and is crucial during viral infection. Although a direct functional connection between the transcription and decay is currently lacking, available data strongly suggest the existence of such interplay which may play a vital role in diverse physiological processes.

## BENEFITS OF COUPLING BETWEEN TRANSCRIPTION AND mRNA DECAY

The functional connection between the synthesis and degradation of mRNAs in eukaryotic cells play a vital physiological role in shaping the characteristic gene expression patterns during diverse cellular processes, such as cell division/cell cycle, developmental program, cell proliferation, cell cycle progression, inflammatory response, cellular response to stress and other environmental cues, and in biological evolution. This interplay is vital to coordinate the gene expression pattern into a rapid and sharp oscillation in the levels of hundreds to thousands of transcripts simultaneously within a very narrow window of time which is essential to respond to such cues. Under such circumstances, the sharp rise in the steady state levels of the mRNA(s) can be more efficiently achieved if the decreased rate of decay kinetics of such transcripts renders a simultaneous and positive feedback to enhance their rate of transcription. Examples for such functional coupling between these two processes shaping appropriate gene expression profile include the regulation of specific and global mRNA steady-state levels during osmotic stress in *S. cerevisiae*, where a small change in the osmolarity in the medium brings about a dramatic up-regulation of a large subset of messages 181. Similar, coordination between the transcription and mRNA decay causes an enhancement of a huge burst of transcripts of induced genes that is concurrent with a dramatic destabilization of the messages of the repressed genes during the mild heat shock and DNA damage response pathways 182,183,184.

Furthermore, the functional linkage between the rate of synthesis and decay provides an efficient way to maintain an appropriate dosage of global transcriptome under a specified physiological condition. Consistent with this view, a recent investigation in *S. cerevisiae* revealed that the cells perfectly and stably maintain a steady and continuous level of diverse transcripts by appropriately modulating their decay rates when the synthesis of the global transcriptome is attenuated by introducing a mutation in the RNAPII 132. Remarkably, these workers also noted that knocking out the catalytic subunit of the Ccr4p/Pop2/Not complex led to a reduced rate of synthesis of global transcripts. Therefore, this observation is consistent with the conclusion that, at least in *S. cerevisiae,* a mutual feedback between these two antagonistic processes is critical for buffering the global level of transcripts 132. Strikingly, this finding also paralleled the previously noted influence of the Δ*dcp1* mutation on enhancing the rate of transcription to maintain an optimal level of cellular transcripts (see above in the previous section) 135. Thus, the ability of the yeast cells to sustain the physiological dosage of global transcripts despite having a defect in any of the transcription or decay machinery strongly implicated a coordinated crosstalk between the mRNA synthesis and decay. However, it remained unclear whether the reduced rate of synthesis of global transcripts is due to reduced rate of transcriptional activity or impaired mRNA decay rate or both.

The characteristic and integrated pattern of gene expression achieved via the interplay between the transcription and the decay kinetics of mRNAs appears to shape the expression profiles of genes during the cell cycle. In *S. cerevisiae*, more than 10% of the protein-coding genes are regulated in a cell cycle-dependent manner 185. Core histone mRNAs provide one such example where entry into the S-phase is accompanied by the rapid increase in their synthesis, followed by a prompt decrease in their abundance as soon as the cells exit the S-phase, presumably by suppressing their transcription and concurrently inducing their decay 186,187. Remarkably, the introduction of an additional copy of histone gene into the haploid yeast cell led to the total increment in the synthesis of the corresponding messages without affecting the steady state mRNA levels, which was presumably balanced by the enhanced mRNA degradation 188. Consistent with this finding, Lsm1-7p was shown to be critical for maintaining the cellular levels of histone mRNAs 189. Similar examples of cell-cycle-dependent temporal control of message abundance involving the cross-talk between the transcription and decay are provided by the control of *SWI5* and *CLB2* mRNAs as mentioned above. Entry into mitosis is associated with the reduction of the abundance of *SWI5* and *CLB2* messages, which is presumed to be the result of a reduced transcription and an increased decay 10. Similar, coupling mechanisms between these two events are postulated to shape the expression levels of distinct gene clusters which undergo cell-cycle-dependent regulation, such as genes involved in DNA synthesis, cytokinesis, budding and other cell cycle-specific events 185,190,191,192,193.

Finally, a functional coupling between transcription and mRNA decay was implicated in promoting the rate of evolution of the organisms. As suggested by Haimovich *et al*., the coordination between the rates of synthesis and decay of messenger RNAs calls for relatively fewer numbers of mutations required to create a unique and desired profile of gene expression to respond to a changing environment 133. Consequently, this unique gene expression profile would lead to the novel and optimum proteome required to achieve one or more distinct phenotypes that would provide the organism with a better selective advantage in the new environment 3. This postulate predicts that only a single regulatory sequence present in the gene (such as a promoter sequence) would be subjected to the evolutionary selection pressure under the new environmental condition(s), thereby demanding the necessity of a fewer number of mutations. Thus, a functional coupling plays a stimulatory role to enhance the rate of evolution, leading to a greater biodiversity 3.

Possible crosstalk between the synthesis of specific sets mRNAs in the nucleus and their regulated degradation appears to shape the expression profiles of the relevant transcriptome in the mammalian system as well during cell differentiation and proliferation, cell-cycle progression, immune and inflammatory response. Strikingly, gamma herpes virus seems to exploit the feedback loop between transcription and mRNA decay to take control of the cellular machinery to shape their gene expression pattern. Future research should unveil (i) more direct evidence of functional crosstalk between transcription and mRNA decay in mammals as well as (ii) the molecular insight of such interplay between these two counterintuitive processes in mammals.

## CONCLUDING REMARKS AND FUTURE PERSPECTIVES

Emerging evidence strongly suggests that a functional coupling exists between the synthesis and decay of mRNAs in yeast, and possibly in mammals. Their coordinated control appears to play key roles in the underlying mechanisms involved in achieving the optimum expression patterns of gene clusters during the cell cycle, cell proliferation, cell cycle progression, immune and inflammatory response, cellular response to stress, drugs, diverse environmental signals, and developmental cues. Such cross-talk between these two opposing physiological processes is thought to contribute to the origin of mRNA regulons in *S. cerevisiae*, clusters of transcripts encoding functionally related proteins, which are co-regulated in a collective fashion during cell growth and differentiation 194,195,196,197. Comparable kind of mRNA regulons were also reported in *Drosophila* and mammals 194, thereby indicating that similar feedback mechanisms may also exist in larger classes of genes/mRNAs in higher organisms. However, the functional coupling between transcription and mRNA degradation are rather difficult to detect and demonstrate experimentally owing to the lack of availability of direct measurement techniques to quantify transcription and decay simultaneously. Notably, some progress in this direction has been made in the recent years 9,10,198,199, which should potentially accelerate a rapid advancement in this field.

This functional interplay between mRNA synthesis and degradation, however, leave us with quite a few interesting, yet unresolved, issues with unanswered questions. Despite a substantial amount of progress in this field, it is still unclear how promoters and transcription factors bring about the decay rates of a specific subset of transcripts. For example, how the specific RNAPII core proteins, Rpb4/7, stimulate the deadenylation of a specific set of transcripts is unclear. Although Rpb4/7 was found to interact with the decay factors (Pat1p, Lsm1-7) and was also found to localize to P-bodies together with them 12,13, the exact functional implications of this interaction are poorly understood for the following reasons.

First, Rpb4/7p was implicated in the deadenylation event 12,13,80 but neither Pat1p or Lsm1p are directly connected to deadenylation 89 nor a direct interaction between Rpb4/7p and the Ccr4p/Pop2/Not /Pan2-3p complex was ever detected 12,13. Thus, the mechanistic insight into the Rpb4/7p induced deadenylation of the PBF mRNAs is not completely understood and requires additional investigations.

Second, how Rpb4/7p is selectively deposited onto the specific sets of messages in a transcription-dependent manner (presumably via Rpb1p-CTD) is not known. Whether this deposition involves modifications of specific residues of Rpb1p-CTD or requires another new coordinator is not known.

Third, comprehensive knowledge about the functional connections between diverse transcriptional aspects and the mRNA decay linked via the Ccr4p/Pop2/Not complex are also incomplete. The principal reason for this lack of information could be accounted by the fact that the roles of the deadenylase complex in transcription and mRNA decay were investigated independently and separately. This gap could be successfully bridged if they are studied together.

Fourth, the exact molecular mechanism of "marking" specific messages or subsets of messages with a given coordinator remains poorly understood. In other words, it is uncertain how the specific transcription factors/coordinators, which influence mRNA decay rates, leave their marks on the specific transcripts for the rest of their life. Nonetheless, a clue to this question was partially addressed by Trcek *et al*., who provided strong evidence that recruitment of Dbf2p, the MEN kinase onto the promoter of *SWI5* and *CLB2* promoter sequence is critical for the stability of these transcripts 10. These authors also showed that subsequent loading of Dbf2p onto the *SWI5* and *CLB2* messages and interaction of Dbf2p with its cytosolic partner, Dbf20p, plays a vital role in forming a cell cycle specific "mark" on the *SWI5* and *CLB2* mRNA. However, how the recruitment of Dbf2p to the promoter sequence influences the stability of these messages is not understood. Interestingly, Dbf2p was found to be part of a larger interactome consisting of the Ccr4p/Pop2p/Not complex, implicating this factor in recruiting the decay components onto the marked message. Thus, Dbf2p appears to link the processes of initial marking of the message with the coordinator and later recruitment of decay factors onto the marked message in a selective fashion. However, the factor(s) critical for marking the *SWI5* and *CLB2* messages is yet to be identified, which also leaves the molecular mechanism of Dbf2/Dbf20 dependent decay of these mRNAs an unresolved issue. Further research is certainly required to answer these questions.

Fifth, how a "mark" present on the specific transcript or a set of messages is later decoded in the cytoplasm is another unresolved issue. A clue to this question came from the studies by Bregman *et al*., who suggested that promoter mediated decay of *RPL30* mRNA involves the deadenylase Ccr4p/Pop2p/Not complex and cytoplasmic exoribonuclease Xrn1p via a 5’→3‘ decay mechanism 9. Therefore, it is logical to suggest that the Ccr4/Not complex is certainly involved in the decoding process. This proposition also received the support from the data presented by Trcek and his colleagues, who showed that Dbf2 also interact with the Ccr4p/Pop2/Not complex 10. What is still unknown in this line is how this mark alters the rate of deadenylation and decapping of the specific subset of messages.

Sixth, it would be quite exciting to uncover the entire plethora of "synthegradases", class of transcription factors which might be involved in coordinating transcription with mRNA decay of other classes of transcripts and also participate in the regulation of this event. Identification of the novel synthegradases can be accomplished by exploring the status of the post-translational modifications (if any) of these transcription factors following their initial identification. Also, the epigenetic requirement/consequences (if any) of the functional coupling between the synthesis and decay of mRNA should be addressed and explored. Particularly, the role of chromatin and other DNA binding proteins in this event (which in turn may govern the regulation of larger subsets of genes) in connection to this question would be very relevant.

While the molecular mechanism involved in the interplay between mRNA synthesis and decay is currently unknown, these studies pointed out that coupling the transcription to decay certainly increases the efficacy of the fine-tuning of the expression of environmentally induced genes 9,10,90,200. This "counteraction", potentially imposed by the "synthegradeses", may play a critical role in the regulation of expression of many mRNAs whose induction was known to occur in stepwise response 182,199. Consistent with this idea, approximately 10% of the yeast genes preserved through evolution were found to employ the functional coupling between transcription and mRNA decay 8,90. Future research using the newer technology enabling simultaneous measurements of the rates of transcription and mRNA-decay is necessary to address what extents of total cellular messages in *S. cerevisiae* exercise this kind of connected circuitry to respond to a wide range of stress, environmental, cell-cycle specific and developmental cues. Furthermore, input from research using other model organisms would also help this field to evaluate the relevance of the interplay between the transcription and mRNA decay, concerning the overall regulation of eukaryotic gene expression with a possible connection to human diseases (if any).

## References

[1] Bjork P, Wieslander L (2014). Mechanisms of mRNA export.. Semin Cell Dev Biol.

[2] Chlebowski A, Lubas M, Jensen TH, Dziembowski A (2013). RNA decay machines: the exosome.. Biochim Biophys Acta.

[3] Haimovich G, Choder M, Singer RH, Trcek T (2013). The fate of the messenger is pre-determined: a new model for regulation of gene expression.. Biochim Biophys Acta.

[4] Nagarajan VK, Jones CI, Newbury SF, Green PJ (2013). XRN 5'→3' exoribonucleases: structure, mechanisms and functions.. Biochim Biophys Acta.

[5] Perez-Ortin JE, Alepuz P, Chavez S, Choder M (2013). Eukaryotic mRNA decay: methodologies, pathways, and links to other stages of gene expression.. J Mol Biol.

[6] Wahle E, Winkler GS (2013). RNA decay machines: deadenylation by the Ccr4-not and Pan2-Pan3 complexes.. Biochim Biophys Acta.

[7] Fasken MB, Corbett AH (2016). Links between mRNA splicing, mRNA quality control, and intellectual disability.. RNA Dis.

[8] Dori-Bachash M, Shalem O, Manor YS, Pilpel Y, Tirosh I (2012). Widespread promoter-mediated coordination of transcription and mRNA degradation.. Genome Biol.

[9] Bregman A, Avraham-Kelbert M, Barkai O, Duek L, Guterman A, Choder M (2011). Promoter elements regulate cytoplasmic mRNA decay.. Cell.

[10] Trcek T, Larson DR, Moldon A, Query CC, Singer RH (2011). Single-molecule mRNA decay measurements reveal promoter- regulated mRNA stability in yeast.. Cell.

[11] Enssle J, Kugler W, Hentze MW, Kulozik AE (1993). Determination of mRNA fate by different RNA polymerase II promoters.. Proc Natl Acad Sci U S A.

[12] Lotan R, Bar-On VG, Harel-Sharvit L, Duek L, Melamed D, Choder M (2005). The RNA polymerase II subunit Rpb4p mediates decay of a specific class of mRNAs.. Genes Dev.

[13] Lotan R, Goler-Baron V, Duek L, Haimovich G, Choder M (2007). The Rpb7p subunit of yeast RNA polymerase II plays roles in the two major cytoplasmic mRNA decay mechanisms.. J Cell Biol.

[14] Harel-Sharvit L, Eldad N, Haimovich G, Barkai O, Duek L, Choder M (2010). RNA polymerase II subunits link transcription and mRNA decay to translation.. Cell.

[15] Luna R, Gaillard H, Gonzalez-Aguilera C, Aguilera A (2008). Biogenesis of mRNPs: integrating different processes in the eukaryotic nucleus.. Chromosoma.

[16] Kohler A, Hurt E (2007). Exporting RNA from the nucleus to the cytoplasm.. Nat Rev Mol Cell Biol.

[17] Vinciguerra P, Stutz F (2004). mRNA export: an assembly line from genes to nuclear pores.. Curr Opin Cell Biol.

[18] Le Hir H, Gatfield D, Izaurralde E, Moore MJ (2001). The exon-exon junction complex provides a binding platform for factors involved in mRNA export and nonsense-mediated mRNA decay.. EMBO J.

[19] Stutz F, Izaurralde E (2003). The interplay of nuclear mRNP assembly, mRNA surveillance and export.. Trends Cell Biol.

[20] Jensen TH, Dower K, Libri D, Rosbash M (2003). Early formation of mRNP: license for export or quality control?. Mol Cell.

[21] Daneholt B (2001). Assembly and transport of a premessenger RNP particle.. Proc Natl Acad Sci U S A.

[22] Muller-McNicoll M, Neugebauer KM (2013). How cells get the message: dynamic assembly and function of mRNA-protein complexes.. Nat Rev Genet.

[23] Proudfoot NJ, Furger A, Dye MJ (2002). Integrating mRNA processing with transcription.. Cell.

[24] Moore MJ, Proudfoot NJ (2009). Pre-mRNA processing reaches back to transcription and ahead to translation.. Cell.

[25] Izaurralde E, Stepinski J, Darzynkiewicz E, Mattaj IW (1992). A cap binding protein that may mediate nuclear export of RNA polymerase II-transcribed RNAs.. J Cell Biol.

[26] Izaurralde E, Lewis J, McGuigan C, Jankowska M, Darzynkiewicz E, Mattaj IW (1994). A nuclear cap binding protein complex involved in pre-mRNA splicing.. Cell.

[27] Reed R, Cheng H (2005). TREX, SR proteins and export of mRNA.. Curr Opin Cell Biol.

[28] Dimaano C, Ullman KS (2004). Nucleocytoplasmic transport: integrating mRNA production and turnover with export through the nuclear pore.. Mol Cell Biol.

[29] Reed R (2003). Coupling transcription, splicing and mRNA export.. Curr Opin Cell Biol.

[30] Tran EJ, Wente SR (2006). Dynamic nuclear pore complexes: life on the edge.. Cell.

[31] Rodriguez-Navarro S, Hurt E (2011). Linking gene regulation to mRNA production and export.. Curr Opin Cell Biol.

[32] Neugebauer KM (2002). On the importance of being co-transcriptional.. J Cell Sci.

[33] Buratowski S (2009). Progression through the RNA polymerase II CTD cycle.. Mol Cell.

[34] Bentley DL (2005). Rules of engagement: co-transcriptional recruitment of pre-mRNA processing factors.. Curr Opin Cell Biol.

[35] Perales R, Bentley D (2009). "Cotranscriptionality": the transcription elongation complex as a nexus for nuclear transactions.. Mol Cell.

[36] Maniatis T, Reed R (2002). An extensive network of coupling among gene expression machines.. Nature.

[37] Rodriguez MS, Dargemont C, Stutz F (2004). Nuclear export of RNA.. Biol Cell.

[38] Bird G, Fong N, Gatlin JC, Farabaugh S, Bentley DL (2005). Ribozyme cleavage reveals connections between mRNA release from the site of transcription and pre-mRNA processing.. Mol Cell.

[39] Aguilera A (2005). Cotranscriptional mRNP assembly: from the DNA to the nuclear pore.. Curr Opin Cell Biol.

[40] Parker R (2012). RNA degradation in Saccharomyces cerevisae.. Genetics.

[41] Klauer AA, van Hoof A (2012). Degradation of mRNAs that lack a stop codon: a decade of nonstop progress.. Wiley Interdiscip Rev RNA.

[42] Ghosh S, Jacobson A (2010). RNA decay modulates gene expression and controls its fidelity.. Wiley Interdiscip Rev RNA.

[43] Neu-Yilik G, Kulozik AE (2008). NMD: multitasking between mRNA surveillance and modulation of gene expression.. Adv Genet.

[44] Schmid M, Jensen TH (2013). Transcription-associated quality control of mRNP.. Biochim Biophys Acta.

[45] Saguez C, Olesen JR, Jensen TH (2005). Formation of export-competent mRNP: escaping nuclear destruction.. Curr Opin Cell Biol.

[46] Anderson JT (2005). RNA turnover: unexpected consequences of being tailed.. Curr Biol.

[47] Schmid M, Jensen TH (2008). Quality control of mRNP in the nucleus.. Chromosoma.

[48] Lykke-Andersen S, Jensen TH (2015). Nonsense-mediated mRNA decay: an intricate machinery that shapes transcriptomes.. Nat Rev Mol Cell Biol.

[49] Lykke-Andersen S, Tomecki R, Jensen TH, Dziembowski A (2011). The eukaryotic RNA exosome: same scaffold but variable catalytic subunits.. RNA Biol.

[50] Schneider C, Tollervey D (2013). Threading the barrel of the RNA exosome.. Trends Biochem Sci.

[51] Tuck AC, Tollervey D (2011). RNA in pieces.. Trends Genet.

[52] Ishigaki Y, Li X, Serin G, Maquat LE (2001). Evidence for a pioneer round of mRNA translation: mRNAs subject to nonsense-mediated decay in mammalian cells are bound by CBP80 and CBP20.. Cell.

[53] Gao Q, Das B, Sherman F, Maquat LE (2005). Cap-binding protein 1-mediated and eukaryotic translation initiation factor 4E-mediated pioneer rounds of translation in yeast.. Proc Natl Acad Sci U S A.

[54] Maquat LE, Hwang J, Sato H, Tang Y (2010). CBP80-promoted mRNP rearrangements during the pioneer round of translation, nonsense-mediated mRNA decay, and thereafter.. Cold Spring Harb Symp Quant Biol.

[55] Nover L, Scharf KD, Neumann D (1983). Formation of cytoplasmic heat shock granules in tomato cell cultures and leaves.. Mol Cell Biol.

[56] Kedersha NL, Gupta M, Li W, Miller I, Anderson P (1999). RNA-binding proteins TIA-1 and TIAR link the phosphorylation of eIF-2 alpha to the assembly of mammalian stress granules.. J Cell Biol.

[57] Sheth U, Parker R (2006). Targeting of aberrant mRNAs to cytoplasmic processing bodies.. Cell.

[58] Balagopal V, Parker R (2009). Polysomes, P bodies and stress granules: states and fates of eukaryotic mRNAs.. Curr Opin Cell Biol.

[59] Beelman CA, Parker R (1994). Differential effects of translational inhibition in cis and in trans on the decay of the unstable yeast MFA2 mRNA.. J Biol Chem.

[60] Beelman CA, Stevens A, Caponigro G, LaGrandeur TE, Hatfield L, Fortner DM, Parker R (1996). An essential component of the decapping enzyme required for normal rates of mRNA turnover.. Nature.

[61] Caponigro G, Parker R (1996). Mechanisms and control of mRNA turnover in Saccharomyces cerevisiae.. Microbiol Rev.

[62] Fasken MB, Corbett AH (2005). Process or perish: quality control in mRNA biogenesis.. Nat Struct Mol Biol.

[63] Houseley J, LaCava J, Tollervey D (2006). RNA-quality control by the exosome.. Nat Rev Mol Cell Biol.

[64] Doma MK, Parker R (2007). RNA quality control in eukaryotes.. Cell.

[65] Davis CA, Ares Jr M (2006). Accumulation of unstable promoter-associated transcripts upon loss of the nuclear exosome subunit Rrp6p in Saccharomyces cerevisiae.. Proc Natl Acad Sci U S A.

[66] Neil H, Malabat C, d'Aubenton-Carafa Y, Xu Z, Steinmetz LM, Jacquier A (2009). Widespread bidirectional promoters are the major source of cryptic transcripts in yeast.. Nature.

[67] Wyers F, Rougemaille M, Badis G, Rousselle JC, Dufour ME, Boulay J, Regnault B, Devaux F, Namane A, Seraphin B, Libri D, Jacquier A (2005). Cryptic pol II transcripts are degraded by a nuclear quality control pathway involving a new poly(A) polymerase.. Cell.

[68] Xu Z, Wei W, Gagneur J, Perocchi F, Clauder-Munster S, Camblong J, Guffanti E, Stutz F, Huber W, Steinmetz LM (2009). Bidirectional promoters generate pervasive transcription in yeast.. Nature.

[69] Chen CY, Shyu AB (2011). Mechanisms of deadenylation-dependent decay.. Wiley Interdiscip Rev RNA.

[70] Schwartz DC, Parker R (2000). mRNA decapping in yeast requires dissociation of the cap binding protein, eukaryotic translation initiation factor 4E.. Mol Cell Biol.

[71] She M, Decker CJ, Sundramurthy K, Liu Y, Chen N, Parker R, Song H (2004). Crystal structure of Dcp1p and its functional implications in mRNA decapping.. Nat Struct Mol Biol.

[72] She M, Decker CJ, Svergun DI, Round A, Chen N, Muhlrad D, Parker R, Song H (2008). Structural basis of dcp2 recognition and activation by dcp1.. Mol Cell.

[73] Coller JM, Tucker M, Sheth U, Valencia-Sanchez MA, Parker R (2001). The DEAD box helicase, Dhh1p, functions in mRNA decapping and interacts with both the decapping and deadenylase complexes.. RNA.

[74] Arribas-Layton M, Wu D, Lykke-Andersen J, Song H (2013). Structural and functional control of the eukaryotic mRNA decapping machinery.. Biochim Biophys Acta.

[75] Poole TL, Stevens A (1995). Comparison of features of the RNase activity of 5'-exonuclease-1 and 5'-exonuclease-2 of Saccharomyces cerevisiae.. Nucleic Acids Symp Ser.

[76] Anderson JS, Parker RP (1998). The 3' to 5' degradation of yeast mRNAs is a general mechanism for mRNA turnover that requires the SKI2 DEVH box protein and 3' to 5' exonucleases of the exosome complex.. EMBO J.

[77] Araki Y, Takahashi S, Kobayashi T, Kajiho H, Hoshino S, Katada T (2001). Ski7p G protein interacts with the exosome and the Ski complex for 3'-to-5' mRNA decay in yeast.. EMBO J.

[78] van Hoof A, Staples RR, Baker RE, Parker R (2000). Function of the ski4p (Csl4p) and Ski7p proteins in 3'-to-5' degradation of mRNA.. Mol Cell Biol.

[79] Liu H, Rodgers ND, Jiao X, Kiledjian M (2002). The scavenger mRNA decapping enzyme DcpS is a member of the HIT family of pyrophosphatases.. EMBO J.

[80] Goler-Baron V, Selitrennik M, Barkai O, Haimovich G, Lotan R, Choder M (2008). Transcription in the nucleus and mRNA decay in the cytoplasm are coupled processes.. Genes Dev.

[81] Kolodziej PA, Woychik N, Liao SM, Young RA (1990). RNA polymerase II subunit composition, stoichiometry, and phosphorylation.. Mol Cell Biol.

[82] Edwards AM, Kane CM, Young RA, Kornberg RD (1991). Two dissociable subunits of yeast RNA polymerase II stimulate the initiation of transcription at a promoter in vitro.. J Biol Chem.

[83] Orlicky SM, Tran PT, Sayre MH, Edwards AM (2001). Dissociable Rpb4-Rpb7 subassembly of rna polymerase II binds to single-strand nucleic acid and mediates a post-recruitment step in transcription initiation.. J Biol Chem.

[84] Runner VM, Podolny V, Buratowski S (2008). The Rpb4 subunit of RNA polymerase II contributes to cotranscriptional recruitment of 3' processing factors.. Mol Cell Biol.

[85] Farago M, Nahari T, Hammel C, Cole CN, Choder M (2003). Rpb4p, a subunit of RNA polymerase II, mediates mRNA export during stress.. Mol Biol Cell.

[86] Selitrennik M, Duek L, Lotan R, Choder M (2006). Nucleocytoplasmic shuttling of the Rpb4p and Rpb7p subunits of Saccharomyces cerevisiae RNA polymerase II by two pathways.. Eukaryot Cell.

[87] Nissan T, Rajyaguru P, She M, Song H, Parker R (2010). Decapping activators in Saccharomyces cerevisiae act by multiple mechanisms.. Mol Cell.

[88] Tharun S, Parker R (2001). Targeting an mRNA for decapping: displacement of translation factors and association of the Lsm1p-7p complex on deadenylated yeast mRNAs.. Mol Cell.

[89] Hatfield L, Beelman CA, Stevens A, Parker R (1996). Mutations in trans-acting factors affecting mRNA decapping in Saccharomyces cerevisiae.. Mol Cell Biol.

[90] Dori-Bachash M, Shema E, Tirosh I (2011). Coupled evolution of transcription and mRNA degradation.. PLoS Biol.

[91] Darieva Z, Bulmer R, Pic-Taylor A, Doris KS, Geymonat M, Sedgwick SG, Morgan BA, Sharrocks AD (2006). Polo kinase controls cell-cycle-dependent transcription by targeting a coactivator protein.. Nature.

[92] Toyn JH, Araki H, Sugino A, Johnston LH (1991). The cell-cycle-regulated budding yeast gene DBF2, encoding a putative protein kinase, has a homologue that is not under cell-cycle control.. Gene.

[93] Liu HY, Toyn JH, Chiang YC, Draper MP, Johnston LH, Denis CL (1997). DBF2, a cell cycle-regulated protein kinase, is physically and functionally associated with the CCR4 transcriptional regulatory complex.. EMBO J.

[94] Cereghino GP, Atencio DP, Saghbini M, Beiner J, Scheffler IE (1995). Glucose-dependent turnover of the mRNAs encoding succinate dehydrogenase peptides in Saccharomyces cerevisiae: sequence elements in the 5' untranslated region of the Ip mRNA play a dominant role.. Mol Biol Cell.

[95] Lombardo A, Cereghino GP, Scheffler IE (1992). Control of mRNA turnover as a mechanism of glucose repression in Saccharomyces cerevisiae.. Mol Cell Biol.

[96] Cereghino GP, Scheffler IE (1996). Genetic analysis of glucose regulation in saccharomyces cerevisiae: control of transcription versus mRNA turnover.. EMBO J.

[97] Munchel SE, Shultzaberger RK, Takizawa N, Weis K (2011). Dynamic profiling of mRNA turnover reveals gene-specific and system-wide regulation of mRNA decay.. Mol Biol Cell.

[98] Dichtl B, Stevens A, Tollervey D (1997). Lithium toxicity in yeast is due to the inhibition of RNA processing enzymes.. EMBO J.

[99] Hedbacker K, Carlson M (2008). SNF1/AMPK pathways in yeast.. Front Biosci.

[100] McCartney RR, Schmidt MC (2001). Regulation of Snf1 kinase.. Activation requires phosphorylation of threonine 210 by an upstream kinase as well as a distinct step mediated by the Snf4 subunit. J Biol Chem.

[101] Portillo F, Mulet JM, Serrano R (2005). A role for the non-phosphorylated form of yeast Snf1: tolerance to toxic cations and activation of potassium transport.. FEBS Lett.

[102] DeRisi JL, Iyer VR, Brown PO (1997). Exploring the metabolic and genetic control of gene expression on a genomic scale.. Science.

[103] Braun KA, Young ET (2014). Coupling mRNA synthesis and decay.. Mol Cell Biol.

[104] Braun KA, Dombek KM, Young ET (2015). Snf1-Dependent Transcription Confers Glucose-Induced Decay upon the mRNA Product.. Mol Cell Biol.

[105] Braun KA, Vaga S, Dombek KM, Fang F, Palmisano S, Aebersold R, Young ET (2014). Phosphoproteomic analysis identifies proteins involved in transcription-coupled mRNA decay as targets of Snf1 signaling.. Sci Signal.

[106] Zhang Y, Kweon HK, Shively C, Kumar A, Andrews PC (2013). Towards systematic discovery of signaling networks in budding yeast filamentous growth stress response using interventional phosphorylation data.. PLoS Comput Biol.

[107] Young ET, Dombek KM, Tachibana C, Ideker T (2003). Multiple pathways are co-regulated by the protein kinase Snf1 and the transcription factors Adr1 and Cat8.. J Biol Chem.

[108] Goldstrohm AC, Hook BA, Seay DJ, Wickens M (2006). PUF proteins bind Pop2p to regulate messenger RNAs.. Nat Struct Mol Biol.

[109] Tadauchi T, Matsumoto K, Herskowitz I, Irie K (2001). Post-transcriptional regulation through the HO 3'-UTR by Mpt5, a yeast homolog of Pumilio and FBF.. EMBO J.

[110] Hata H, Mitsui H, Liu H, Bai Y, Denis CL, Shimizu Y, Sakai A (1998). Dhh1p, a putative RNA helicase, associates with the general transcription factors Pop2p and Ccr4p from Saccharomyces cerevisiae.. Genetics.

[111] Chen J, Chiang YC, Denis CL (2002). CCR4, a 3'-5' poly(A) RNA and ssDNA exonuclease, is the catalytic component of the cytoplasmic deadenylase.. EMBO J.

[112] Daugeron MC, Mauxion F, Seraphin B (2001). The yeast POP2 gene encodes a nuclease involved in mRNA deadenylation.. Nucleic Acids Res.

[113] Tucker M, Staples RR, Valencia-Sanchez MA, Muhlrad D, Parker R (2002). Ccr4p is the catalytic subunit of a Ccr4p/Pop2p/Notp mRNA deadenylase complex in Saccharomyces cerevisiae.. EMBO J.

[114] Tucker M, Valencia-Sanchez MA, Staples RR, Chen J, Denis CL, Parker R (2001). The transcription factor associated Ccr4 and Caf1 proteins are components of the major cytoplasmic mRNA deadenylase in Saccharomyces cerevisiae.. Cell.

[115] Clark LB, Viswanathan P, Quigley G, Chiang YC, McMahon JS, Yao G, Chen J, Nelsbach A, Denis CL (2004). Systematic mutagenesis of the leucine-rich repeat (LRR) domain of CCR4 reveals specific sites for binding to CAF1 and a separate critical role for the LRR in CCR4 deadenylase activity.. J Biol Chem.

[116] Denis CL (1984). Identification of new genes involved in the regulation of yeast alcohol dehydrogenase II.. Genetics.

[117] Draper MP, Liu HY, Nelsbach AH, Mosley SP, Denis CL (1994). CCR4 is a glucose-regulated transcription factor whose leucine-rich repeat binds several proteins important for placing CCR4 in its proper promoter context.. Mol Cell Biol.

[118] Collart MA, Struhl K (1994). NOT1(CDC39), NOT2(CDC36), NOT3, and NOT4 encode a global-negative regulator of transcription that differentially affects TATA-element utilization.. Genes Dev.

[119] Collart MA (1996). The NOT, SPT3, and MOT1 genes functionally interact to regulate transcription at core promoters.. Mol Cell Biol.

[120] Oberholzer U, Collart MA (1998). Characterization of NOT5 that encodes a new component of the Not protein complex.. Gene.

[121] Miller JE, Reese JC (2012). Ccr4-Not complex: the control freak of eukaryotic cells.. Crit Rev Biochem Mol Biol.

[122] Collart MA, Panasenko OO (2012). The Ccr4--not complex.. Gene.

[123] Reese JC (2013). The control of elongation by the yeast Ccr4-not complex.. Biochim Biophys Acta.

[124] Denis CL, Chiang YC, Cui Y, Chen J (2001). Genetic evidence supports a role for the yeast CCR4-NOT complex in transcriptional elongation.. Genetics.

[125] Biswas D, Yu Y, Mitra D, Stillman DJ (2006). Genetic interactions between Nhp6 and Gcn5 with Mot1 and the Ccr4-Not complex that regulate binding of TATA-binding protein in Saccharomyces cerevisiae.. Genetics.

[126] Kruk JA, Dutta A, Fu J, Gilmour DS, Reese JC (2011). The multifunctional Ccr4-Not complex directly promotes transcription elongation.. Genes Dev.

[127] van Dijk E, Cougot N, Meyer S, Babajko S, Wahle E, Seraphin B (2002). Human Dcp2: a catalytically active mRNA decapping enzyme located in specific cytoplasmic structures.. EMBO J.

[128] Steiger M, Carr-Schmid A, Schwartz DC, Kiledjian M, Parker R (2003). Analysis of recombinant yeast decapping enzyme.. RNA.

[129] Grousl T, Ivanov P, Frydlova I, Vasicova P, Janda F, Vojtova J, Malinska K, Malcova I, Novakova L, Janoskova D, Valasek L, Hasek J (2009). Robust heat shock induces eIF2alpha-phosphorylation-independent assembly of stress granules containing eIF3 and 40S ribosomal subunits in budding yeast, Saccharomyces cerevisiae.. J Cell Sci.

[130] Gaudon C, Chambon P, Losson R (1999). Role of the essential yeast protein PSU1 in p6anscriptional enhancement by the ligand-dependent activation function AF-2 of nuclear receptors.. EMBO J.

[131] Shalem O, Groisman B, Choder M, Dahan O, Pilpel Y (2011). Transcriptome kinetics is governed by a genome-wide coupling of mRNA production and degradation: a role for RNA Pol II.. PLoS Genet.

[132] Sun M, Schwalb B, Pirkl N, Maier KC, Schenk A, Failmezger H, Tresch A, Cramer P (2013). Global analysis of eukaryotic mRNA degradation reveals Xrn1-dependent buffering of transcript levels.. Mol Cell.

[133] Haimovich G, Medina DA, Causse SZ, Garber M, Millan-Zambrano G, Barkai O, Chavez S, Perez-Ortin JE, Darzacq X, Choder M (2013). Gene expression is circular: factors for mRNA degradation also foster mRNA synthesis.. Cell.

[134] Kearsey S, Kipling D (1991). Recombination and RNA processing: a common strand?. Trends Cell Biol.

[135] Muhlrad D, Parker R (1999). Recognition of yeast mRNAs as "nonsense containing" leads to both inhibition of mRNA translation and mRNA degradation: implications for the control of mRNA decapping.. Mol Biol Cell.

[136] Schwartz D, Decker CJ, Parker R (2003). The enhancer of decapping proteins, Edc1p and Edc2p, bind RNA and stimulate the activity of the decapping enzyme.. RNA.

[137] Sanduja S, Blanco FF, Dixon DA (2011). The roles of TTP and BRF proteins in regulated mRNA decay.. Wiley Interdiscip Rev RNA.

[138] Gherzi R, Chen CY, Trabucchi M, Ramos A, Briata P (2010). The role of KSRP in mRNA decay and microRNA precursor maturation.. Wiley Interdiscip Rev RNA.

[139] Winkler GS (2010). The mammalian anti-proliferative BTG/Tob protein family.. J Cell Physiol.

[140] Mauxion F, Chen CY, Seraphin B, Shyu AB (2009). BTG/TOB factors impact deadenylases.. Trends Biochem Sci.

[141] Matsuda S, Rouault J, Magaud J, Berthet C (2001). In search of a function for the TIS21/PC3/BTG1/TOB family.. FEBS Lett.

[142] Tirone F (2001). The gene PC3(TIS21/BTG2), prototype member of the PC3/BTG/TOB family: regulator in control of cell growth, differentiation, and DNA repair?. J Cell Physiol.

[143] Ito Y, Suzuki T, Yoshida H, Tomoda C, Uruno T, Takamura Y, Miya A, Kobayashi K, Matsuzuka F, Kuma K, Yamamoto T, Miyauchi A (2005). Phosphorylation and inactivation of Tob contributes to the progression of papillary carcinoma of the thyroid.. Cancer Lett.

[144] Iwanaga K, Sueoka N, Sato A, Sakuragi T, Sakao Y, Tominaga M, Suzuki T, Yoshida Y, J KT, Yamamoto T, Hayashi S, Nagasawa K, Sueoka E (2003). Alteration of expression or phosphorylation status of tob, a novel tumor suppressor gene product, is an early event in lung cancer.. Cancer Lett.

[145] Yoneda M, Suzuki T, Nakamura T, Ajima R, Yoshida Y, Kakuta S, Katsuko S, Iwakura Y, Shibutani M, Mitsumori K, Yokota J, Yamamoto T (2009). Deficiency of antiproliferative family protein Ana correlates with development of lung adenocarcinoma.. Cancer Sci.

[146] Kawakubo H, Brachtel E, Hayashida T, Yeo G, Kish J, Muzikansky A, Walden PD, Maheswaran S (2006). Loss of B-cell translocation gene-2 in estrogen receptor-positive breast carcinoma is associated with tumor grade and overexpression of cyclin d1 protein.. Cancer Res.

[147] Guehenneux F, Duret L, Callanan MB, Bouhas R, Hayette S, Berthet C, Samarut C, Rimokh R, Birot AM, Wang Q, Magaud JP, Rouault JP (1997). Cloning of the mouse BTG3 gene and definition of a new gene family (the BTG family) involved in the negative control of the cell cycle.. Leukemia.

[148] Yoshida Y, Matsuda S, Ikematsu N, Kawamura-Tsuzuku J, Inazawa J, Umemori H, Yamamoto T (1998). ANA, a novel member of Tob/BTG1 family, is expressed in the ventricular zone of the developing central nervous system.. Oncogene.

[149] Ikematsu N, Yoshida Y, Kawamura-Tsuzuku J, Ohsugi M, Onda M, Hirai M, Fujimoto J, Yamamoto T (1999). Tob2, a novel anti-proliferative Tob/BTG1 family member, associates with a component of the CCR4 transcriptional regulatory complex capable of binding cyclin-dependent kinases.. Oncogene.

[150] Buanne P, Corrente G, Micheli L, Palena A, Lavia P, Spadafora C, Lakshmana MK, Rinaldi A, Banfi S, Quarto M, Bulfone A, Tirone F (2000). Cloning of PC3B, a novel member of the PC3/BTG/TOB family of growth inhibitory genes, highly expressed in the olfactory epithelium.. Genomics.

[151] Prevot D, Voeltzel T, Birot AM, Morel AP, Rostan MC, Magaud JP, Corbo L (2000). The leukemia-associated protein Btg1 and the p53-regulated protein Btg2 interact with the homeoprotein Hoxb9 and enhance its transcriptional activation.. J Biol Chem.

[152] Busson M, Carazo A, Seyer P, Grandemange S, Casas F, Pessemesse L, Rouault JP, Wrutniak-Cabello C, Cabello G (2005). Coactivation of nuclear receptors and myogenic factors induces the major BTG1 influence on muscle differentiation.. Oncogene.

[153] Ou YH, Chung PH, Hsu FF, Sun TP, Chang WY, Shieh SY (2007). The candidate tumor suppressor BTG3 is a transcriptional target of p53 that inhibits E2F1.. EMBO J.

[154] Tzachanis D, Freeman GJ, Hirano N, van Puijenbroek AA, Delfs MW, Berezovskaya A, Nadler LM, Boussiotis VA (2001). Tob is a negative regulator of activation that is expressed in anergic and quiescent T cells.. Nat Immunol.

[155] Rouault JP, Prevot D, Berthet C, Birot AM, Billaud M, Magaud JP, Corbo L (1998). Interaction of BTG1 and p53-regulated BTG2 gene products with mCaf1, the murine homolog of a component of the yeast CCR4 transcriptional regulatory complex.. J Biol Chem.

[156] Prevot D, Morel AP, Voeltzel T, Rostan MC, Rimokh R, Magaud JP, Corbo L (2001). Relationships of the antiproliferative proteins BTG1 and BTG2 with CAF1, the human homolog of a component of the yeast CCR4 transcriptional complex: involvement in estrogen receptor alpha signaling pathway.. J Biol Chem.

[157] Aslam A, Mittal S, Koch F, Andrau JC, Winkler GS (2009). The Ccr4-NOT deadenylase subunits CNOT7 and CNOT8 have overlapping roles and modulate cell proliferation.. Mol Biol Cell.

[158] Yoshida Y, Hosoda E, Nakamura T, Yamamoto T (2001). Association of ANA, a member of the antiproliferative Tob family proteins, with a Caf1 component of the CCR4 transcriptional regulatory complex.. Jpn J Cancer Res.

[159] Collart MA, Timmers HT (2004). The eukaryotic Ccr4-not complex: a regulatory platform integrating mRNA metabolism with cellular signaling pathways?. Prog Nucleic Acid Res Mol Biol.

[160] Goldstrohm AC, Wickens M (2008). Multifunctional deadenylase complexes diversify mRNA control.. Nat Rev Mol Cell Biol.

[161] Ezzeddine N, Chang TC, Zhu W, Yamashita A, Chen CY, Zhong Z, Yamashita Y, Zheng D, Shyu AB (2007). Human TOB, an antiproliferative transcription factor, is a poly(A)-binding protein-dependent positive regulator of cytoplasmic mRNA deadenylation.. Mol Cell Biol.

[162] Funakoshi Y, Doi Y, Hosoda N, Uchida N, Osawa M, Shimada I, Tsujimoto M, Suzuki T, Katada T, Hoshino S (2007). Mechanism of mRNA deadenylation: evidence for a molecular interplay between translation termination factor eRF3 and mRNA deadenylases.. Genes Dev.

[163] Cougot N, Babajko S, Seraphin B (2004). Cytoplasmic foci are sites of mRNA decay in human cells.. J Cell Biol.

[164] Lopez de Silanes I, Quesada MP, Esteller M (2007). Aberrant regulation of messenger RNA 3'-untranslated region in human cancer.. Cell Oncol.

[165] Benjamin D, Moroni C (2007). mRNA stability and cancer: an emerging link?. Expert Opin Biol Ther.

[166] Liang J, Lei T, Song Y, Yanes N, Qi Y, Fu M (2009). RNA-destabilizing factor tristetraprolin negatively regulates NF-kappaB signaling.. J Biol Chem.

[167] Schichl YM, Resch U, Hofer-Warbinek R, de Martin R (2009). Tristetraprolin impairs NF-kappaB/p65 nuclear translocation.. J Biol Chem.

[168] Ashburner BP, Westerheide SD, Baldwin Jr AS (2001). The p65 (RelA) subunit of NF-kappaB interacts with the histone deacetylase (HDAC) corepressors HDAC1 and HDAC2 to negatively regulate gene expression.. Mol Cell Biol.

[169] Davis-Smyth T, Duncan RC, Zheng T, Michelotti G, Levens D (1996). The far upstream element-binding proteins comprise an ancient family of single-strand DNA-binding transactivators.. J Biol Chem.

[170] Chen CY, Gherzi R, Ong SE, Chan EL, Raijmakers R, Pruijn GJ, Stoecklin G, Moroni C, Mann M, Karin M (2001). AU binding proteins recruit the exosome to degrade ARE-containing mRNAs.. Cell.

[171] Chou CF, Mulky A, Maitra S, Lin WJ, Gherzi R, Kappes J, Chen CY (2006). Tethering KSRP, a decay-promoting AU-rich element-binding protein, to mRNAs elicits mRNA decay.. Mol Cell Biol.

[172] Gherzi R, Lee KY, Briata P, Wegmuller D, Moroni C, Karin M, Chen CY (2004). A KH domain RNA binding protein, KSRP, promotes ARE-directed mRNA turnover by recruiting the degradation machinery.. Mol Cell.

[173] Briata P, Forcales SV, Ponassi M, Corte G, Chen CY, Karin M, Puri PL, Gherzi R (2005). p38-dependent phosphorylation of the mRNA decay-promoting factor KSRP controls the stability of select myogenic transcripts.. Mol Cell.

[174] Gherzi R, Trabucchi M, Ponassi M, Ruggiero T, Corte G, Moroni C, Chen CY, Khabar KS, Andersen JS, Briata P (2006). The RNA-binding protein KSRP promotes decay of beta-catenin mRNA and is inactivated by PI3K-AKT signaling.. PLoS Biol.

[175] Diaz-Moreno I, Hollingworth D, Frenkiel TA, Kelly G, Martin S, Howell S, Garcia-Mayoral M, Gherzi R, Briata P, Ramos A (2009). Phosphorylation-mediated unfolding of a KH domain regulates KSRP localization via 14-3-3 binding.. Nat Struct Mol Biol.

[176] Trabucchi M, Briata P, Garcia-Mayoral M, Haase AD, Filipowicz W, Ramos A, Gherzi R, Rosenfeld MG (2009). The RNA-binding protein KSRP promotes the biogenesis of a subset of microRNAs.. Nature.

[177] Ruggiero T, Trabucchi M, De Santa F, Zupo S, Harfe BD, McManus MT, Rosenfeld MG, Briata P, Gherzi R (2009). LPS induces KH-type splicing regulatory protein-dependent processing of microRNA-155 precursors in macrophages.. FASEB J.

[178] Abernathy E, Gilbertson S, Alla R, Glaunsinger B (2015). Viral Nucleases Induce an mRNA Degradation-Transcription Feedback Loop in Mammalian Cells.. Cell Host Microbe.

[179] Kwong AD, Frenkel N (1987). Herpes simplex virus-infected cells contain a function(s) that destabilizes both host and viral mRNAs.. Proc Natl Acad Sci U S A.

[180] Gaglia MM, Covarrubias S, Wong W, Glaunsinger BA (2012). A common strategy for host RNA degradation by divergent viruses.. J Virol.

[181] Romero-Santacreu L, Moreno J, Perez-Ortin JE, Alepuz P (2009). Specific and global regulation of mRNA stability during osmotic stress in Saccharomyces cerevisiae.. RNA.

[182] Shalem O, Dahan O, Levo M, Martinez MR, Furman I, Segal E, Pilpel Y (2008). Transient transcriptional responses to stress are generated by opposing effects of mRNA production and degradation.. Mol Syst Biol.

[183] Castells-Roca L, Garcia-Martinez J, Moreno J, Herrero E, Belli G, Perez-Ortin JE (2011). Heat shock response in yeast involves changes in both transcription rates and mRNA stabilities.. PLoS One.

[184] Tirosh I (2011). Transcriptional priming of cytoplasmic post-transcriptional regulation.. Transcription.

[185] Spellman PT, Sherlock G, Zhang MQ, Iyer VR, Anders K, Eisen MB, Brown PO, Botstein D, Futcher B (1998). Comprehensive identification of cell cycle-regulated genes of the yeast Saccharomyces cerevisiae by microarray hybridization.. Mol Biol Cell.

[186] Hereford LM, Osley MA, Ludwig 2nd TR, McLaughlin CS (1981). Cell-cycle regulation of yeast histone mRNA.. Cell.

[187] Marzluff WF, Wagner EJ, Duronio RJ (2008). Metabolism and regulation of canonical histone mRNAs: life without a poly(A) tail.. Nat Rev Genet.

[188] Osley MA, Hereford LM (1981). Yeast histone genes show dosage compensation.. Cell.

[189] Herrero AB, Moreno S (2011). Lsm1 promotes genomic stability by controlling histone mRNA decay.. EMBO J.

[190] Wittenberg C, Reed SI (2005). Cell cycle-dependent transcription in yeast: promoters, transcription factors, and transcriptomes.. Oncogene.

[191] Lew DJ (2000). Cell-cycle checkpoints that ensure coordination between nuclear and cytoplasmic events in Saccharomyces cerevisiae.. Curr Opin Genet Dev.

[192] Niu W, Li Z, Zhan W, Iyer VR, Marcotte EM (2008). Mechanisms of cell cycle control revealed by a systematic and quantitative overexpression screen in S.. cerevisiae. PLoS Genet.

[193] Sopko R, Huang D, Preston N, Chua G, Papp B, Kafadar K, Snyder M, Oliver SG, Cyert M, Hughes TR, Boone C, Andrews B (2006). Mapping pathways and phenotypes by systematic gene overexpression.. Mol Cell.

[194] Keene JD (2007). RNA regulons: coordination of post-transcriptional events.. Nat Rev Genet.

[195] Gerber AP, Herschlag D, Brown PO (2004). Extensive association of functionally and cytotopically related mRNAs with Puf family RNA-binding proteins in yeast.. PLoS Biol.

[196] Olivas W, Parker R (2000). The Puf3 protein is a transcript-specific regulator of mRNA degradation in yeast.. EMBO J.

[197] Saint-Georges Y, Garcia M, Delaveau T, Jourdren L, Le Crom S, Lemoine S, Tanty V, Devaux F, Jacq C (2008). Yeast mitochondrial biogenesis: a role for the PUF RNA-binding protein Puf3p in mRNA localization.. PLoS One.

[198] Sun M, Schwalb B, Schulz D, Pirkl N, Etzold S, Larviriere L, Maier KC, Seizl M, Tresch A, Cramer P (2012). Comparative dynamic transcriptome analysis (cDTA) reveals mutual feedback between mRNA synthesis and degradation.. Genome Res.

[199] Rabani M, Levin JZ, Fan L, Adiconis X, Raychowdhury R, Garber M, Gnirke A, Nusbaum C, Hacohen N, Friedman N, Amit I, Regev A (2011). Metabolic labeling of RNA uncovers principles of RNA production and degradation dynamics in mammalian cells.. Nat Biotechnol.

[200] Bellofatto V, Wilusz J (2011). Transcription and mRNA stability: parental guidance suggested.. Cell.

